# Calcifediol (25OH Vitamin D_3_) Deficiency: A Risk Factor from Early to Old Age

**DOI:** 10.3390/nu14061168

**Published:** 2022-03-10

**Authors:** Roger Bouillon, Leen Antonio, Oscar Rosero Olarte

**Affiliations:** 1 Clinical and Experimental Endocrinology, Department of Chronic Diseases and Metabolism, Catholic University of Leuven, 3000 Leuven, Belgium; leen.antonio@kuleuven.be; 2 Department of Endocrinology, University Hospitals Leuven, 3000 Leuven, Belgium; 3 Clinical Endocrinology, Asociación Colombiana de Osteoporosis, Bogotá 500005, Colombia; oscarroseromd@yahoo.com

**Keywords:** vitamin D, calcifediol, osteoporosis, rickets, osteomalacia, immunology, COVID-19

## Abstract

Vitamin D deficiency is the main cause of nutritional rickets in children and osteomalacia in adults. There is consensus that nutritional access to vitamin D can be estimated by measuring serum concentrations of 25OHD and vitamin D deficiency can thus be considered as calcifediol deficiency. However, the threshold for vitamin D/calcifediol sufficiency remains a matter of debate. Vitamin D/calcifediol deficiency has been associated with musculoskeletal effects but also multiple adverse extra-skeletal consequences. If these consequences improve or if they can be treated with vitamin D supplementation is still unclear. Observational studies suggest a higher infection risk in people with low calcifediol levels. There is also a consistent association between serum calcifediol and cardiovascular events and deaths, but large-scale, long-term intervention studies did not show any benefit on cardiovascular outcomes from supplementation, at least not in subjects without clear vitamin D deficiency. Cancer risk also did not change with vitamin D treatment, although there are some data that higher serum calcifediol is associated with longer survival in cancer patients. In pregnant women, vitamin D supplementation decreases the risk of pre-eclampsia, gestational diabetes mellitus, and low birth weight. Although preclinical studies showed that the vitamin D endocrine system plays a role in certain neural cells as well as brain structure and function, there is no evidence to support a beneficial effect of vitamin D in neurodegenerative diseases. Vitamin D supplementation may marginally affect overall mortality risk especially in elderly subjects with low serum calcifediol concentrations.

## 1. Introduction

Vitamin D was discovered a century ago and it has been a topic of intensive basic research and more lately of hundreds of clinical studies, all exploring the consequences of vitamin D status on bone and extra-skeletal health. The lay press has also paid intensive attention to these questions and it has even generated a real hype about the possible benefits of more widespread vitamin D supplementation.

In this manuscript of the special issue of NUTRIENTS, we want to address two questions:

1. Should we relabel what we up till now usually described as “vitamin D deficiency” as “calcifediol deficiency”?
2. What are the clinical consequences of such “calcifediol deficiency”? Should we taper down the hype while avoiding minimizing the consequences of “vitamin D/calcifediol” deficiency?

## 2. History

Endemic rickets was recognized as a severe disease of children in the mid-17th century as reviewed recently [1,2,3,4,5]. Indeed, during that century, Daniel Whistler presented a short (approximately eight pages) overview of the main characteristics of this disease at the University of Leiden (The Netherlands). His description mentioned epiphysial widening, craniotabes, rachitic rosary, bending of the legs, and defective dentition. This publication was followed in London, a few years later, by a very extensive description of the disease by Francis Glisson (in collaboration with G. Bate and A. Regemorter). However, these authors were joined in describing this disease by John Mayow (working in London) and Arnold Boot, a Dutch physician working in Ireland who clearly described the main characteristics of the disease: thoracic malformations and a swelling of the joints in mostly young children (below the age of 3 years). There were many speculations about the etiology of the disease such as the influence of “bad air”; the poor nutrition of the child, the mother, or foster mother; poor blood circulation; and the possible involvement of liver enlargement [4]. The disease was most frequently found in the children of poor families living in cities with poor housing and a lack of exposure to sunlight. It was however also found in some rich families in England and later on also in India (with hindsight due to lack or avoidance of sunshine). The disease was not known or certainly not common in ancient Egypt or the Middle East. It was, however, present in Roman times as proven by careful examination of skeletons from cemeteries in different areas of the Roman empire: a few percent of the skeletons show signs of active or cured rickets in children, adolescents, or adults. The highest frequency (approximately 7%) is found in skeletons of pre-adults buried in Ostea, the “industrialized” port of Rome, known for its high rise buildings and narrow streets [6].

The main characteristics of rickets are age dependent and they are due to insufficient mineralization of bones or growth plates (Table 1) [7,8,9]. We now know that rickets has a variety of etiologies as reviewed by Carpenter [8] and in a WHO report [9]. In essence, the disease can be due to calcium and vitamin D-related problems, phosphate deficiency, or more rare disorders involving genes and proteins related to the mineralization process itself (Table 2). Nutritional rickets can be due to severe calcium deficiency. Thresholds for such deficiency are age dependent and roughly defined by calcium intake below 200–260 mg/d for infants up to 6 or 12 months, respectively, and below 300 mg/d for older infants.

The main cause of nutritional rickets is vitamin D deficiency [7,10]. This is due to a combination of poor nutritional intake of vitamin D and a lack of exposure to sufficient UVB light. Indeed, the vitamin D content of human milk is low, even from mothers living in equatorial areas of the world as the combined vitamin D activity (vitamin D and 25OHD) is usually well below 1 ug/L [10,11]. This is well below the minimal requirements of the total vitamin D needed to prevent rickets. Indeed, 10 ug or 400 IU/d is recommended by most experts; although from clinical experience during the early 20th century, it may well be that 200 IU/d would be sufficient for most infants as 200 IU was recommended by the WHO as the amount needed to prevent rickets and it seemed to be quite efficient.

Since the discovery of vitamin D, we have learned that vitamin D itself is inactive and requires two additional hydroxylations: one at carbon 25 and the other at carbon 1 to generate first 25-hydroxyvitamin D (25OHD or calcifediol) and subsequently 1α,25(OH)_2_D or calcitriol [12]. These hydroxylations are absolutely essential as demonstrated by mutations of these genes in humans and animals. Deficiency of the 1α-hydroxylation of 25OHD by biallelic silencing mutations of CYP27B1 is known as pseudodeficiency rickets, vitamin D dependency rickets, or OMIM #264700. This disease has been described in humans and some domestic or pet animals, and it has been created by genetic engineering in mice [13]. The phenotype is very similar in all species and it is overlapping with the signs and symptoms of nutritional rickets. The disease can be perfectly cured by a daily intake of 1α-hydroxylated vitamin D metabolites (approximately 1 ug/d of 1,25(OH)_2_D in adults). Unlike 1α-hydroxylation of 25OHD, which is due to a single gene and protein, the 25-hydroxylation of vitamin D can be performed by several CYP450 enzymes, but CYP2R1 is clearly the major enzyme. It is mainly but not exclusively expressed in liver, and it is characterized by its high affinity and low capacity. It does not discriminate between vitamin D_2_ and vitamin D_3_. Mutations in CYP2R1 have been found to cause rickets in children from different countries [14]. These children have low but detectable serum concentrations of 25OHD and 1,25(OH)_2_D, and they respond to either high doses of vitamin D or physiologic doses of oral 25OHD [15]. A mouse model of *Cyp2r1* deficiency, however, did not cause rickets although their serum 25OHD was substantially lower than in control mice [16]. However, these mice received a diet with a relatively high calcium content, and no experiments with lower calcium intake have been published to date.

## 3. Vitamin D or Calcifediol Deficiency?

From the observations summarized above, we can conclude that a lack of vitamin D or of vitamin D action causes rickets. We also know that approximately 10 ug of vitamin D per day can prevent this disease in infants and children, and most probably also in adults, at least when intestinal absorption is normal and vitamin D metabolism is intact. How can we now define vitamin D deficiency? Insufficient vitamin D intake is not a valid criterium as most vitamin D is synthesized in the skin. Nutritional intake of vitamin D is variable and difficult to measure, and this is even more so for endogenous vitamin D synthesis after UVB exposure. The serum concentration of vitamin D itself is not a good parameter of overall access to vitamin D as it only reflects very recent exposure. Indeed, vitamin D disappears rapidly from circulation by uptake in the liver and possibly other tissues. Serum 1,25(OH)_2_D is also not a good reflection of nutritional access to vitamin D as it has a short half-life and it is tightly regulated by ions and hormones. As vitamin D of whatever origin is rapidly converted into 25OHD and as 25OHD has a long half-life (approximately 2 weeks) and it is the obligatory precursor of the active hormone, there is general consensus that serum concentrations of 25OHD are the best parameter to estimate nutritional access to vitamin D. Based on several clinical studies, serum concentrations of 25OHD below 10 or 12 ng/mL, and especially below 5 ng/mL, are a risk factor for rickets (or osteomalacia). This threshold is not absolute as it is based on older studies using less accurate assays. Recent data suggest that the threshold is higher in combination with poor calcium intake [17]. If vitamin D deficiency is in fact defined by serum concentrations of 25OHD, we could call it “calcifediol deficiency”. Even with a clear clinical or radiological picture of rickets, we still measure serum 25OHD as proof of nutritional deficiency as its etiology, excluding other causes of this disease.

In conclusion, nutritional rickets is due to a lack of vitamin D (action) before closure of the growth plate. There are several reasons why one could argue that such vitamin D deficiency could also be relabeled as calcifediol deficiency:

1. The obligatory intermediate 25OHD is between vitamin D and the active hormone, 1,25(OH)_2_D;
2. Nutritional vitamin D deficiency implies calcifediol deficiency;
3. Mutations in CYP2R1 result in clinical rickets (also called vitamin D-dependent rickets) and this can be corrected by low (physiologic) doses of calcifediol, whereas a normal supply of vitamin D cannot cure this disease;
4. Serum concentrations of 25OHD are the best marker of nutritional vitamin D deficiency. Low serum calcifediol is thus the hallmark of this deficiency and used for confirmation of the diagnosis or for the estimation of risk of this disease in a population.

Actions to prevent calcifediol deficiency

1. Measure serum 25OHD using accurate methods in subjects with nutritional rickets as to better define the threshold and relative risk of low vitamin D status;
2. Implement vitamin D supplementation for all children below the age of 3 years and even more so in infants during their first year of life;
3. Implement minimal dietary calcium in the diets of infants in countries or regions of the world with usually low calcium intake.

## 4. Consequences of Vitamin D or Calcifediol Deficiency

### Musculoskeletal Effects

Vitamin D action is essential for bone homeostasis as severe deficiency impairs mineral deposition at any age. This is characterized by increased osteoid thickness and surface, increased surface of osteoclastic bone resorption, and most importantly, a decreased mineral apposition rate and a delayed mineral deposition lag time. Unfortunately, these criteria require bone histomorphometry, preferably allowing measurement of dynamic parameters by prior in vivo bone labeling techniques. There are indeed at present no validated imaging techniques to define osteomalacia except in the case of pseudofractures or Milkman–Looser lines. Bone mineral density measurements cannot distinguish between osteoporosis and osteomalacia. Before the closure of the growth plate, the most easily identifiable characteristics of a lack of vitamin D or a lack of vitamin D action are abnormal growth plates, as described above. There are radiological criteria that allow the estimation of the severity of rickets [7]. The vitamin D hormone has effects on many bone cells. Exposure to 1,25(OH)_2_D regulates several osteoblast genes including the typical osteoblast marker, osteocalcin. This also includes genes involved in the communication between osteoblasts and osteoclasts [12]. Additionally, 1,25(OH)_2_D is one of most potent agents able to stimulate osteoclast formation although this action is mainly mediated by its effect on osteoblasts or immune cells. Whether vitamin D action is essential for bone is a matter of dispute. A total lack of VDR or CYP27B1 causes rickets but this is mainly due to its effect on intestinal calcium absorption as shown by the correction of bone and growth plate structure by a rescue diet of high calcium and lactose. Cell-specific deletion of *Vdr* or *Cyp27b1* allows for the evaluation of the direct effects of vitamin D action on bone or growth plates. Osteoblast or osteocyte specific *Vdr* null mice have either a normal bone phenotype or they display mild increases in bone mass; *Vdr* or *Cyp27B1* deletion in chondrocytes creates a transient phenotype of modestly increased bone density [18]. Surprisingly, overexpression of *Vdr* or 1,25(OH)_2_D in osteoblasts also modestly increases bone mass. Therefore, it is difficult to define the precise direct effect of 1,25(OH)_2_D on bone as it may depend on the stage of bone development or the species. However, overall, the direct effects are relatively minor in comparison with the systemic effects. Indeed, the primary non-redundant target tissue for the action of the vitamin D endocrine system is the intestinal mucosa. This has been shown by selective deletion of *Vdr* in intestinal cells (whether proximal or more distal parts of the intestinal track) causing rickets. Conversely, total *Vdr* null mice with intestinal-specific rescue of *Vdr* in the intestine restores a normal bone/growth plate phenotype. The action of vitamin D-calcifediol-1,25(OH)_2_D on the intestine is mainly the stimulation of calcium transport by regulating a number of genes such as TRPV6 at the mucosal membrane, intracellular calcium binding proteins (such as calbindin 9k), or serosal calcium transporters. In addition, the VDR endocrine system regulates tight junction genes (such as claudins) thereby mediating paracellular calcium transport [19,20].

As revealed by low calcifediol concentrations, the consequences of poor vitamin D status during puberty, adulthood, and old age—other than osteomalacia—is somewhat disputed and it is the topic of another manuscript in this special issue of NUTRIENTS [21]. In essence, most experts believe that poor vitamin D status (not sufficiently deficient to cause osteomalacia) can stimulate secondary hyperparathyroidism and negatively influence the balance between bone formation and resorption, thereby accelerating osteoporosis and bone fragility fractures. Apart from many observational studies suggesting such a link, intervention studies have shown a beneficial effect of vitamin D supplementation of elderly subjects with calcifediol deficiency (as demonstrated by low serum 25OHD) on BMD and fractures (whether hip fractures, major fractures, or all fractures combined) [21,22,23]. An additional question is whether vitamin D status may influence the fracture healing process. As the vitamin D endocrine system regulates a very large number of genes, it is no surprise that a substantial number of these genes are also involved in the different stages of fracture healing, starting from the stage of inflammation, callus formation, bone formation, and remodeling [24]. In addition, 24R,25(Oh)2D has been shown to have a remarkable effect on callus formation and fracture healing in an animal model of *Cyp24a1* null mice. Indeed, 24R,25(OH)2D is able to bind to a specific membrane receptor, FAM57B2, followed by stimulation of the formation of lactosylceramide and thereby stimulate callus formation [25]. Observational studies in humans have generated contradictory results. There are only a few randomized controlled trials dealing with the effect of vitamin D (usually combined with calcium) supplementation on fracture healing. These small-scale studies did not generate clear answers, but they suggest that correction of vitamin D deficiency may improve the late stages of fracture healing [26]. A controlled study however could not demonstrate a radiological advantage of vitamin D supplementation for elderly subjects with vertebral fractures [27]. In line with these data, a new small-scale study showed no effect on bone characteristics after a low bolus dose of vitamin D, whereas a higher dose suggested even a negative effect on bone mineral density or strength [28]. Therefore, it is plausible that severe vitamin D deficiency could impair optimal bone mineralization of a healing fracture, but there is no consensus about the benefits of vitamin D for fracture healing in patients with modest vitamin D status.

In conclusion, the intestinal mucosa is a well-defined, non-redundant target tissue for the action of vitamin D-calcifediol-1,25(OH)_2_D-VDR, especially by stimulating the absorption of calcium. Although many genes and most bone cells are targets of the vitamin D endocrine system, the direct effect of this system is small in comparison with the indirect effect mediated by intestinal calcium absorption. Severe calcifediol deficiency (below 12 ng/mL) generates a risk for rickets or osteomalacia, whereas more modest calcifediol deficiency (serum concentrations between 12 and 20 ng/mL) likely shifts the balance of bone homeostasis in favor of greater bone resorption compared to bone formation, thereby accelerating age-related bone loss.

## 5. Extra-Skeletal Consequences of Calcifediol Deficiency

In view of the widespread presence of VDR and CYP27B1 and the very large number of genes under control of the vitamin D endocrine system (VDES), virtually every system of the body has been listed as a potential target. Here we will review the major systems or tissues for which there are sufficient good data to formulate the best answer or at least the best “educated guess”.

### 5.1. The Immune System

All the cells of the immune system express VDR at a certain stage of their life cycle and a very large number of genes involved in the immune system are under the control of the VDES. This is an early event in the evolution of vertebrates as many “immune” genes are regulated by the injection of 1,25(OH)_2_D in young zebra fish [29]. Overall, the native immune system (mainly represented by monocytes and macrophages) is activated by 1,25(OH)_2_D, as reviewed elsewhere in this special issue of NUTRIENTS [30]. This action should decrease the risk of infections in subjects receiving sufficient vitamin D either naturally or by supplementation. Observational studies indeed confirm that vitamin D deficiency is frequently associated with infections such as HIV, tuberculosis, or upper respiratory infections. Large meta-analyses of vitamin D supplementation trials also confirmed a reduction in the risk of upper respiratory infections, especially when given on a regular basis (not in bolus doses) to subjects with a poor vitamin D status [31]. Unfortunately, a large RCT did not reduce the risk of tuberculosis in Mongolian children with calcifediol deficiency [32]. Obviously, the possible beneficial effects of good vitamin D status on the risk or the severity of COVID-19 infection has attracted a lot of attention with hundreds of publications since its first description. Overall, observational data suggest that the risk of infection may be slightly higher in the case of low calcifediol concentration, but there are no good randomized controlled trials. There are stronger arguments that calcifediol supplementation shortly before or after the onset of the infection decreases the severity of the disease as evaluated by the subsequent need for ICU treatment or by overall mortality. These data are the topic of one of the manuscripts of the present special issue of NUTRIENTS [33]. By contrast, the acquired immune system is downregulated by 1,25(OH)_2_D. Indeed, the major cell type of the acquired immune system (dendritic cells) changes in morphology and function when exposed to 1,25(OH)_2_D, thereby decreasing the activation of T helper 1-17-21 cells and upregulating the T helper 2 cells and the T regulatory cells. This action is confirmed in many animal models. NOD mice (prone to autoimmune diabetes) have a higher risk of diabetes when born to vitamin D deficient mothers [34,35]. Treatment of mice with 1,25(OH)_2_D or especially with vitamin D analogs with lower calcemic effects, can decrease the risk or severity of autoimmune diabetes, glomerulonephritis, or experimental autoimmune encephalitis (= animal model of multiple sclerosis) [36]. Observational studies have repeatedly found lower levels of calcifediol in subjects with multiple sclerosis, autoimmune diabetes, or other autoimmune diseases. Unfortunately, there are no major invention studies confirming the beneficial effects of treatment with vitamin D or its analogs for the prevention of autoimmune diseases. Mendelian randomization (MR) studies allow evaluation of lifelong genetically predisposed lower vitamin D status (as revealed by gene polymorphisms associated with lower serum concentrations of calcifediol). Four independent MR studies clearly indicated a higher risk of multiple sclerosis (whether childhood or adult onset) in subjects genetically predisposed to lower calcifediol levels [23].

In summary, the immune system is a plausible target for actions of the VDES as based on extensive preclinical data. Human intervention studies suggest a causal link between vitamin D/calcifediol status and infections, whereas MR studies strongly suggest a link between lifelong lower calcifediol levels and the risk of multiple sclerosis.

### 5.2. Cardiovascular System

Vitamin D null mice have high serum concentrations of renin, angiotensin II, and aldosterone. This can be explained by high renin concentrations in their kidneys as the renin gene is under the negative control of 1,25(OH)_2_D. Therefore, they develop high renin hypertension and subsequently cardiac hypertrophy and heart muscle fibrosis. The same phenotype is also found in mice with *Cyp27b1* deficiency and in vitamin D deficient animals [37,38]. The causality is further confirmed as the problem cannot be solved by a high calcium rescue diet (unlike the bone phenotype), but it can be successfully treated by angiotensin blockers or 1,25(OH)_2_D supplementation of *Cyp27B1* deficient animals, excluding *Vdr* null mice. Cardiac myocyte hypertrophy and cardiac fibrosis is also found in vitamin D deficient animals [39]. A direct effect on cardiac myocyte is also likely as cardiac muscle specific *Vdr* null mice also develop cardiac hypertrophy and fibrosis [39]. Moreover, 1,25(OH)_2_D stimulates calcium uptake by cardiomyocytes, and it increases their contractility and relaxation [37].

The VDES also has a complex action of the vasculature by its effects on endothelial and smooth muscle cells; 1,25(OH)_2_D stimulates prostaglandins and thus vascular wall relaxation; and, similarly, 1,25(OH)_2_D stimulates endothelial nitric oxide synthase (eNOS) in aortic tissue. Indeed, expression of eNOS was reduced by 50% in aortic tissue from *Vdr* global knock-out mice on a rescue diet in comparison to wild-type controls [40]. It also inhibits thrombogenesis and increased fibrinolysis as the opposite is observed in *Vdr* null mice [41]. The VDES also has a complex effect on many cytokines produced by cells of the vascular wall thereby exhibiting anti-inflammatory effects. Mice with selective deletion of *Vdr* in monocytes show increased atheromatosis [42] and this is also observed in mice with an overexpression of *Cyp24a1* (which accelerates the degradation of 1,25(OH)_2_D). This is in line with the observation of decreased uptake of cholesterol by macrophages and decreased oxidized LDL incorporation into atheroma lesions. All these preclinical data strongly suggest a rather beneficial spectrum of activities of the VDES on the cardiovascular system. There is however at least one caveat as 1,25(OH)_2_D can stimulate the transdifferentiation of vascular smooth muscle cells into osteoblast-like cells able to express typical osteoblast genes and deposit calcium in the extracellular matrix, thereby calcifying the vascular wall. This calcification is however not the same as atherosclerosis but nevertheless impairs the flexibility of the vascular wall [37,38].

There is a wealth of human observational data associating poor vitamin D status (based on low serum calcifediol concentrations) and cardiovascular risk factors and cardiovascular events. Cross sectional studies frequently found lower serum calcifediol levels in subjects with cardiovascular events [37]. More importantly, longitudinal studies confirmed such trends. In the Framingham Heart Study, using stored serum samples, subjects with serum 25OHD concentrations below 15 ng/mL had a 60% higher risk of a cardiovascular event over a 7-year time period, and this remained highly significant even after adjustment for other risk factors [43]. In the Health Professionals Follow-up Study, men with serum 25OHD levels below 15 ng/mL also had a twofold increased risk of myocardial infarction compared to men with a better calcifediol status. This also applied to mortality as an endpoint as Finnish men in the highest quintile of serum calcifediol had a 24% lower adjusted risk of cardiovascular death. Several NHANES studies confirmed a consistent link between low serum 25OHD and cardiovascular events, especially cardiovascular death (+40%) [44,45].

In an overview of eight meta-analyses dealing with observational studies, lower serum 25OHD was linked with all kinds of cardiovascular events, ranging from hypertension, ischemic stroke, and myocardial infarction to cardiovascular death [38]. This conclusion is thus based on observational data from several hundred thousand subjects, including long-term prospective studies. The risk is not linear as there seems to be a plateau effect when serum calcifediol exceeds 25 ng/mL [46].

Observational studies however should be considered as hypothesis-generating and not as final proof of causality. Mendelian randomization may indicate the lifelong consequences of genetically predisposed lower serum calcifediol concentrations. Six MR studies based on two to six SNPs predicting approximately a five percent variation in serum calcifediol in more than a million subjects found no effect of calcifediol status on cardiovascular events or mortality. This was confirmed in studies using a much larger number (>200) of SNPs, allowing for the evaluation of a greater predicted difference in serum 25OHD [23]. These studies do not fully exclude a relationship between cardiovascular events and calcifediol status as MR cannot evaluate non-linear relationships (as predicted by observational studies). Moreover, even the MR studies dealing with a large number of SNPs cannot predict more than a 10% variation in serum calcifediol and thus cannot predict the lifelong consequences of more severe vitamin D deficiency. However, a UK Biobank study combining measurements of serum calcifediol and MR revealed that genetically low serum calcifediol and low measured serum calcifediol increased overall, including cardiovascular mortality [47].

Intervention studies should generate the final answer (Table 3). Older studies were not designed to look at cardiovascular endpoints as primary or secondary outcome criteria. The Women’s Health Initiative trial studied more than 36,000 women and it could not find a reduced risk of stroke or coronary events in the combined calcium and vitamin D supplementation arm of the trial [48]. Similarly, a British trial using an intermittent dose of 100,000 IU of vitamin D every 4 months revealed a non-significant relative risk (0.84; CI 0.65–1.10) of cardiovascular mortality in a post hoc analysis [49]. In the RECORD trial, a daily dose of 800 IU of vitamin D reduced the risk of heart failure but not of stroke or other cardiovascular events [50]. More recently, results from large-scale, long-term studies have looked at cardiovascular endpoints as primary or secondary outcomes. In the VITAL study more than 25,000 adults were randomized to either vitamin D (2000 IU/d) or placebo for a mean duration of 5.3 years and no effects on cardiovascular events (either individual outcomes or combined cardiovascular events) were found [51]. Similarly, in the New Zealand ViDA trial, a monthly dose of 100,000 IU of vitamin D did not change any cardiovascular event [52]. In the D2d study (evaluating the effects of vitamin D supplementation to prevent progress to type 2 diabetes, subjects with prediabetes were randomized or not to a daily dose of 4000 IU of vitamin D for a mean duration of 2.5 years. No significant effects were observed in major cardiovascular endpoints despite the fact that the subjects, being prediabetic and mostly overweight, were at a higher-than-normal cardiovascular risk. However, there was a small beneficial effect on cardiovascular risk factors in the vitamin D supplemented group [53]. A substudy of the ViDA trial looked at the effect of vitamin D supplementation on blood pressure measured by invasive technology. Peripheral blood pressure did not change but central systolic pressure and six other parameters slightly, but significantly, improved in subjects with a baseline serum calciferol level below 20 ng/mL [54].

The large RCTs, therefore, did not generate clear cardiovascular benefits but most of the subjects in these three major RCTs were vitamin D replete at baseline. Therefore, the subanalysis of subjects with the lowest calcifediol status at baseline was underpowered to answer whether correction of vitamin D deficiency may improve cardiovascular risks or events. A meta-analysis of these studies using the original patient data may help to answer that important question. It is also reassuring that even high doses of vitamin D did not create harmful cardiovascular side effects as experimental vitamin D toxicity in animals causes diffuse vascular calcifications, sometimes even lethal.

### 5.3. Cell Proliferation and Cancer

One of the first extra-skeletal effects of 1,25(OH)_2_D was the observation of an in vitro effect of 1,25(OH)_2_D on the cell proliferation of cancer cells. Indeed, Colston et al. and Abe et al. [56,57] demonstrated a clear inhibition of cell proliferation of myeloma or melanoma cells, respectively, when exposed to 1,25(OH)_2_D. This was rapidly confirmed in most normal cells and many cancer cells. The mechanism behind this inhibitory effect on cell proliferation is due to its effect on several clusters of genes such as cyclin dependent kinase inhibitors (positive effects on p17 and 21), cyclins and cyclin kinase (inhibitory effects), c-myc expression, and retinoblastoma phosphorylation, ultimately resulting in Go/G1 cell cycle arrest. In addition, it regulates prostaglandin synthesis and prostaglandin action. As 1,25(OH)_2_D can also decrease the activity of growth factors such as IGF1 (by upregulation of IFGBP3), it inhibits epidermal growth factor expression and increases TGFβ. Other mechanisms include modulation of intracellular kinase pathways (such as p38MAPK, ERK, and PI3K) or several miRNAs. In certain cells, it also interferes with wnt signaling, favoring lower proliferation and induction of differentiation. Indeed, 1,25(OH)_2_D can also stimulate the differentiation of many cells using cell type specific pathways. In some circumstances it also stimulates apoptosis, decreases angiogenesis, and decreases mechanisms involved in tissue invasion and metastasis. Therefore, 1,25(OH)_2_D displays a variety of mechanisms, without a clear central master key gene or protein, all contributing to a decrease in cell proliferation and stimulation of cell differentiation. Such an action profile is expected to decrease the risk of cancer. This has extensively been tested in preclinical models. First, administration of pharmacological doses of 1,25(OH)_2_D or its less calcemic analogs is able to decrease the growth of many cancers but usually without total separation of their anticancer and calcemic effects [58,59]. The *Vdr* null mice do not show a spontaneous increase in cancer but *Cyp27b1* mice, kept until old age in a Chinese laboratory, display an increased rate of cancers [60]. Even more importantly, the *Vdr* null mice exposed to other events predisposing to cancer such as exposure to chemocarcinogens, oncogenes, or to loss of anti-oncogenes have an increased rate of cancer. Similarly, *Vdr* null mice exposed to UVB light develop more skin cancer than control mice [22,61]. The results in animals thus clearly indicate a possible link between vitamin D action and cancer, but such experiments were performed in animals with either a total lack of vitamin D action or after exposure to very high doses of 1,25(OH)_2_D or its analogs. Such extreme conditions are very exceptional in humans, but a large percentage of humans are coping with a rather poor vitamin D status as reflected by low serum concentrations of calcifediol.

Some but not all observational studies have found an association between calcifediol deficiency and cancer risk, either in cross sectional studies or in long-term prospective studies. This association was especially present for colorectal cancers, whereas for most other cancers the data have been mixed. Using a different approach, several studies have evaluated the prognosis (including cancer mortality) and serum calcifediol levels before or at the time of diagnosis of cancer. Some observational studies reported that higher serum 25OHD concentrations were associated with longer survival among cancer patients [57]. Based on 16 RCTs, there is some evidence that use of vitamin D supplementation improved survival of patients with known cancers but not in the general population [62,63]. In an overview of 10 large RCTs no consistent effects (neither positive nor negative) were found [22]. Only a post hoc subanalysis revealed a tendency for lower colorectal cancer or breast cancer risk in women not taking hormone therapy (WHI trial) and they did not take calcium and vitamin D supplementation before the start of the trial [22]. Two large RCTs have evaluated the potential beneficial effects of vitamin D supplementation on cancer as a primary endpoint. In the ViDA trial, 100,000 IU of vitamin D per month did not change the risk of cancer in general or in any subtype. This was also the conclusion of the larger VITAL trial and the D2d study [64]. Very recently, the results of the Australian D Health trial were published. Slightly more than 21,000 adults (>60 years) were randomized into placebo or a monthly 60,000 IU dose of vitamin D for a mean of 5.7 years of follow up. The primary end point of all-cause mortality revealed no benefit (HR of 1.04, CL 0.93–1.18). Similarly, no effect was seen on cardiovascular or cancer mortality (88).

A different question is whether supplementation may have an influence on the survival of patients. The ViDA trial did not find an effect on cancer mortality but the VITAL trial found a trend for lower cancer mortality [51]. When several trials were combined, a modestly significant lower cancer mortality was found in the subjects receiving vitamin D supplements [65].

In summary, biochemical and genetic data strongly support a possible role of 1,25(OH)_2_D on cell cycle control and tumor growth. The absence of vitamin action by VDR mutations also enhances the development of a variety of cancers either at older age or when exposed to other factors that increase cancer risks such as exposure to chemocarcinogenic agents, UVB light, or genes involved in carcinogenesis. Some but not all observational data suggest a link between poor vitamin D status and cancer incidence, especially for colorectal cancers. Most MR studies however do not confirm a link between lifelong lower vitamin D status and a risk of cancer. Similarly, the existing intervention studies so far could not demonstrate a clear benefit of vitamin D supplementation on the incidence of major human cancers (Table 4). Supplementation may however have a modest effect on cancer mortality. Therefore, supplementation cannot be recommended for the sole purpose of primary or secondary cancer prevention.

## 6. Reproduction

### 6.1. Vitamin D and Fertility

The vitamin D metabolizing enzymes and the VDR are expressed in the human testis, epididymis, seminal vesicle (SV), prostate, and spermatozoa. These vitamin D-related genes are predominantly expressed in the germ cells of testes from adults, which indicate that local regulation of active vitamin D may be important for spermatogenesis and/or sperm function. In VDR KO mice, CYP19A1 activity was 58% and 38% lower in testis and epididymis, respectively, compared to wild-type animals [70,71]. The reduction in estrogen levels was partly reversed by calcium supplementation. Nevertheless, 1,25(OH)_2_D can regulate aromatase activity by direct action on VDRE elements in the aromatase promotor. Strangely, aromatase activity was stimulated in testicular tissue and inhibited in breast tissue [71]. Vitamin D deficient rats or mice with either *Vdr* or *Cyp27b1* KO show a reduced fertility in males but this can be corrected by correcting calcium homeostasis, thereby suggesting that the VDES is redundant for male fertility [72]. Low serum 25OHD concentrations have been associated with lower sperm counts but there are no data that vitamin D supplementation can improve male reproduction [72].

Similar to male reproduction, there are a few studies suggesting a possible involvement of the VDES in female reproduction. The strongest association is between low serum 25OHD concentrations and polycystic ovary syndrome. That disease is however strongly linked with obesity and insulin resistance and it may thus only reflect an association without causality. Indeed, vitamin D supplementation has not been able to change BMI or PCOS [73].

### 6.2. Vitamin D and Pregnancy Outcome

Many pregnant women around the world have rather low serum calcifediol concentrations and this has been associated with an increased risk for the health of the mother, the fetus/neonate, and even on later health events of the offspring. Mean serum 25OHD below 10 ng/mL was found in more than 10% of pregnant women in most Mediterranean countries [74] and approximately half of pregnant women living in Mongolia [75]. Similar results were found in many other cross-sectional studies. An overview of 54 observational studies from around the world concluded that mothers with serum 25OHD concentrations below 12 ng/mL had a higher risk of lower birth weight (mean difference—88 g) [76] and a higher risk of babies that were small for gestational age (OR 1.59), and such risk remained higher (OR 1.43) in women with serum 25OHD concentrations below 20 ng/mL compared with women with higher serum calcifediol levels. The health implications can of course be best evaluated by randomized controlled trials. Such intervention studies were summarized in a Cochrane review [77]. These authors updated their analysis in 2019, evaluating 22 RCTs and 3725 women, with essentially the same conclusions. This meta-analysis concluded that vitamin D supplementation reduced the risk of pre-eclampsia (RR 0.48), gestational diabetes mellitus (RR 0.51), and low birth weight (RR 0.55) (all statistically significant) [77]. Another meta-analysis evaluated 24 RCTs dealing with 5405 participants and it concluded that vitamin D supplementation decreased the risk of being small-for-gestational age (OR significant at 0.72) but not for perinatal mortality. Vitamin D supplementation slightly increased the mean birth weight by 75g [78]. A large RCT in Bangladesh dealing with mostly severely vitamin D deficient mothers (mean serum 25OHD of 10 ng/mL), however, did not find a beneficial effect of vitamin D supplementation starting at around 20 weeks of gestation on their offspring either at birth or at age 1 (no effects of body length, weight, or head circumference) [79]. Wagner et al. evaluated the effects of large doses of vitamin D starting before 16 weeks of gestation and they found a decrease in complications of pregnancy and rates of cesarian section [80,81]. A study in India where most mothers had modest vitamin D deficiency revealed that vitamin D supplementation decreased the combined risk of preterm labor/pre-eclampsia and gestational diabetes by approximately 50% [82]. Other studies looked at the very long-term consequences of the vitamin D status of the mother on the health of their offspring. Four prospective studies and three RCTs concluded that vitamin D supplementation (~800 IU/day) during pregnancy is inversely related to wheezing or asthma in their offspring during up to 3 years of follow-up. However, a longer follow-up did not confirm this conclusion as vitamin D supplementation during the prenatal period alone did not influence the 6-year incidence of asthma and recurrent wheeze among children at risk of asthma.

## 7. Brain

The vitamin D receptor has been detected in the cortex, amygdala, thalamus, and hippocampus [83]. There is evidence that 1,25(OH)_2_D can also be synthesized in the central nervous system as the 1α-hydroxylase has been found in neurons and glia. Several genes of neurons, astrocytes, and glia cells are under the control of 1,25(OH)_2_D. In addition, CYP24A1 is expressed in some brain cells, indicating a mechanism for local feedback control of vitamin D actions [84]. Therefore, it is plausible that the VDES may play a role in brain development and synaptic plasticity [85]. Animal models have shown that maternal hypovitaminosis D induces changes in brain structure and function [86], which subsequently affects memory and learning processes [86,87]. Indeed, maternal hypovitaminosis D induces lasting changes in the brain, which can lead to alterations in brain architecture at birth, although the changes observed in vitamin D deficient rats were somewhat different from those observed in vitamin D deficient mice [85,86,87]. There are many “brain” genes under the control of the VDES (including nerve growth factor), but it seems that it may play a specific role in the differentiation of dopaminergic neurons [88]. The VDES in the nervous system may also participate in the regulation of calcium-mediated neuronal excitotoxicity in the reduction of oxidative stress and in the induction of synaptic structural proteins, neurotrophic factors, and expression of neurotransmitters [89]. Unfortunately, so far no mice with neuron or glia cell specific deletions of *Vdr* or *Cyp27b1* have been reported [86].

Low serum levels of calcifediol have been found in patients with Alzheimer’s or Parkinson’s disease, multiple sclerosis, autism spectrum disorders, sleep disorders, and schizophrenia [90]. The causality of these associations however has not been proven in randomized controlled trials or MR studies. Therapy and reverse causality therefore remain a possible scenario.

## 8. Mortality

If a good calcifediol status would have an (even small) effect on many extra-skeletal consequences, it may well be that this results in a lower mortality rate. Most original studies dealing with musculoskeletal end points also looked at mortality as a safety parameter. Meta-analyses of such studies revealed a small decrease (less than 10%) in overall mortality with a confidence interval that just did or did not include 1 [22]. Although such small effect would be rather trivial for individual subjects, it may have more remarkable benefits at a population level. In a long-term (12 years) follow-up of the EMAS ageing male study of 1915 subjects, the mortality risk was twice as high for males with serum calcifediol levels below 20 ng/mL [91], when compared with subjects with better vitamin D status. The UK Biobank data were used to prospectively study a possible link between calcifediol status and all-cause or cause-specific mortality of 365,530 participants who had serum calcifediol measurements but no history of cardiovascular disease (CVD), cancer, or diabetes at baseline. During a follow up of approximately 9 years, 10,175 deaths occurred. The multivariate analyses revealed a nonlinear inverse association between mortality and serum 25OHD. Subjects with a baseline 25OHD above 24 ng/mL had a significantly 17% lower risk of all-cause mortality (Hazard Ratio of 0.83) and a 23% lower risk of cardiovascular mortality, whereas subjects with serum 25OHD above 18 ng/mL had a 11% lower risk of cancer mortality [92]. The authors conclude that RCTs are needed to confirm such conclusions. They also suggest that calcifediol thresholds of 18–24 ng/mL may be useful to guide clinicians in dosing vitamin D supplementation (trials).

The large VITAL and ViDA studies of course also evaluated the consequences of vitamin D supplementation on mortality. No effect was found for all-cause or cardiovascular mortality. Also in the recent D-Health trial there was no reduction in all-cause mortality with monthly vitamin D supplementation in unscreened subjects 60 years or older [93]. As for the ViDA and VITAL trial, the Australian D health study subjects were mostly vitamin D replete at baseline as mean serum 25OHD was approximately 30 ng/mL in the control group, increasing to 45 ng/mL in the supplemented group. The ViDA trial also did not find an effect on cancer mortality whereas the larger VITAL trial showed a marginally significant lower cancer mortality in subjects receiving vitamin D (2000 IU/d), especially when deaths during the first 2 years of follow up were excluded. This effect was significant from 4 years of supplementation onwards [51]. In the D-Health trial, on the other hand, exploratory analysis showed some evidence of an increased risk of death from cancer in the treatment arm, after exclusion of deaths in the first 2 years of follow-up. As mentioned in the section on cardiovascular events, a meta-analysis of several such long-term studies confirmed a modest but significant reduction in cancer mortality [51,93,94].

Apart from RCTs, MR studies allow estimation of the effects of lower calcifediol levels, as predicted from a variety of gene polymorphisms. No significant effect of genetically lower 25OHD concentrations on overall or cause-specific mortality was found in several studies summarized in a recent review [23]. However, combining a large MR study (*n* = 386,406) with real measurements of calcifediol (*n* = 500,962) [47] confirmed:

(1) Lower serum calcifediol concentrations are linked with increased overall or cause-specific (cardiovascular or cancer) mortality;
(2) Genetically lower serum calcifediol did not change mortality in the overall study group;
(3) Genetically lower serum calcifediol increased overall cardiovascular (both significantly) and cancer (just not significant) mortality in the subgroup with measured low-serum calcifediol (below 10 ng/mL).

## 9. Conclusions

UVB irradiation of 7-dehydrocholesterol is the origin of all vitamin D. Most human vitamin D comes from this kind of synthesis in the skin, whereas a small fraction comes from dietary intake. Vitamin D is an inactive precursor and needs first a hydroxylation at position 25 to generate 25OHD or calcifediol. This is the obligatory intermediate and substrate for CYP27B1 as to generate 1,25(OH)_2_D or calcitriol. Calcitriol binds to its receptor, VDR; and, it regulates a very large number of genes, most of which are not involved in calcium, mineral, or bone homeostasis. A lack of vitamin D or vitamin D action results in rickets (before closure of the growth plates) or osteomalacia. A poor vitamin D status likely also aggravates an imbalance between bone formation and resorption, thereby accelerating osteoporosis and fragility fractures. The best marker of vitamin D status is measurement of serum calcifediol. Therefore, calcifediol deficiency is the biochemical marker of vitamin D deficiency or vitamin D status in general. Deficiency in calcifediol may also have implications for extra-skeletal health. Preclinical studies, including biochemical, genetic, cellular, and animal data all strongly suggest that total absence of vitamin D action has implications for the immune, metabolic, or cardiovascular systems as well as for cell proliferation, cancer, and other health problems. Most but not all observational studies also suggest that a poor calcifediol status is associated with all these extra-skeletal health effects. Older, mostly small-scale supplementation studies generated mixed results. However, recent large-scale randomized controlled trials do not confirm that vitamin D supplementation has major extra-skeletal health effects. Indeed, vitamin D supplementation did not have beneficial effects on the risk of cardiovascular events, development of type 2 diabetes, or cancer; but, most of the study subjects were vitamin D replete at baseline. Vitamin D supplementation, however, seems to decrease the risk of upper respiratory infections (mainly in subjects with a poor calcifediol status) and to decrease the risk of cancer mortality. The MR studies also repeatedly confirmed that subjects with genetic predisposition for lower calcifediol levels have a higher risk of developing juvenile or adult-onset multiple sclerosis. Based on a combination of a large number of RCTs, it seems that vitamin D supplementation studies may also modestly decrease overall mortality and possibly some cause-specific (cardiovascular or cancer) mortality.

## Figures and Tables

**Table 1:** Main characteristics of nutritional rickets.

Early Signs
Delayed fontanel closure (normally closed by age 2 years)
Craniotabes (softening of skull bones, best detected by palpation of cranial sutures in first 3 months)
Bone pain
Restlessness and irritability
Swelling of wrists and ankles
Frontal bossing
Rachitic rosary (enlarged costochondral joints)
**Late signs**
Delayed tooth eruption (no incisors by age 10 months, no molars by age 18 months)
Leg deformity (genu varum, genu valgum)
**Osseous/Radiographic features**
Splaying, fraying, cupping, and coarse trabecular pattern of metaphyses
Widening of the growth plates
Osteopenia
Pelvic deformities including outlet narrowing (risk of obstructed labour and death)
Long-term or permanent deformities of childhood abnormalities of bone
Minimal trauma fracture
**Non-osseous features**
Hypocalcemic seizure and tetany
Hypocalcemic dilated cardiomyopathy (heart failure, arrhythmia, cardiac arrest, death)
Failure to thrive and poor linear growth
Delayed gross motor development with muscle weakness
Raised intracranial pressure

**Table 2:** Main etiologies of rickets.

Calcium Related
*Calcium deficiency with normal or low-normal vitamin D status*
Very low dietary calcium intake
Calcium malabsorption (similar causes as vitamin D malabsorption, see below)
**Vitamin D-related**
*Severe vitamin D deficiency*
Low sunshine exposure and low dietary intake
Malabsorption (e.g., after bariatric surgery, bowel resection, celiac disease, cholestatic liver disease, exocrine pancreatic insufficiency, inflammatory bowel disease)
Increased renal loss of vitamin D and 25OHD (nephrotic syndrome)
Increased catabolism: especially drug-induced or genetic mutations of CYP3A4
Impaired hepatic 25-hydroxylation: mostly due to genetic mutations of CYP2R1 (OMIM #600081)
Impaired renal 1α-hydroxylation: chronic kidney disease (renal osteomalacia), or genetic (=1α-hydroxylase (CYP27B1) deficiency (OMIM #264700)
*Vitamin D resistant rickets*
Hereditary vitamin D-resistant rickets (VDR mutations) (OMIM #277440)
Vitamin D-dependent rickets with normal VDR (hnRNP overexpression) (OMIM #600785)
**Phosphate related rickets/osteomalacia or Hypophosphatemic rickets/osteomalacia**
*Gastrointestinal causes*
Poor nutritional intake (e.g., breastfed very low birth weight infants),
Chronic diarrhea
Excessive phosphate binders
*Renal phosphate wasting*
- Tumor-induced (oncogenic) osteomalacia - Fanconi syndrome (mostly HIV medications such as tenofovir) - X-linked dominant hypophosphatemic rickets (PHEX mutations) (OMIM #307800) - X-linked recessive hypophosphatemic rickets (CLCN5 mutations) (OMIM #300554) - Autosomal dominant hypophosphatemic rickets (FGF23 mutations) (OMIM #193100) - Autosomal recessive hypophosphatemic rickets type 1 (DMP1 mutations) (OMIM #241520), type 2 (ENPP1 mutations) (OMIM #613312) - Hereditary hypophosphatemic rickets with hypercalciuria (SLC34A3) (OMIM #241530) - Dent disease-1 (CLCN5 mutations) (OMIM #300009,) - Dent disease-2 (OCRL mutations) (OMIM #300555)
**Rickets and osteomalacia related to inhibition of mineralization**
Metabolic acidosis (genetic or acquired)
Metal related: aluminum toxicity (e.g., from antacids, dialysis fluid), fluorosis, iron, cadmium, strontium, etc.
Hypophosphatasia (inorganic pyrophosphate accumulation) (OMIM #146300)
Matrix abnormalities Type VI osteogenesis imperfecta (SERPINF1 mutations) (OMIM #613982)
Fibrogenesis imperfecta ossium
Axial osteomalacia

**Table 3:** Intervention studies with vitamin D supplementation and cardiovascular events.

See REF.	Study, Number of Subjects	Follow-Up	Treatment	Outcome
Hsia [48]	original WHI trial *n* = 36,282	7 years	400 IU	HR for—MI or coronary heart disease death: 1.04—Stroke: 0.95
Bolland [55]	WHI reanalysis *n* = 16,718 women not taking calcium supplements at baseline	7 years	400 IU D3 + 1 g calcium	HR (all NS) for: —MI: 1.2 —coronary revascularization: 1.15 —Stroke: 1.17 —All CV events: 1.13
Ford [50]	Record trial *n* = 5292	9 years	800 IU D3 + 1 g calcium	HR for —cardiac failure: 0.75 * —MI: 0.97 —Stroke: 1.06
Scragg [52]	VIDA trial (New Zealand) *n* = 5108	3.3 years	100,000 IU D3 per month (baseline mean 25OHD: 24 ng/mL)	HR for -all CV diseases: 1.02
Manson [51]	VITAL Trial *n* = 25,871	5.3 years	2000 IU D3/d	HR for MACE 0.97 (0.85–1.11) All cause of mortality: 0.99 (0.87–1.11) Cardiovascular Death 1.10 (0.88–1.39)

WHI: Women’s Health Initiative; RECORD trial: randomized evaluation of calcium or vitamin D; VIDA: Vitamin D Assessment study; VITAL: VITamin D and OmegA-3 TriaL; HR: hazard ratio; CV: cardiovascular; MACE: major adverse cardiovascular events; REF: reference; *: HR for —cardiac failure: 0.75.

**Table 4:** Meta-analyses of Vitamin D Supplementation and Prevention of Cancer.

REF.	Number of Subjects	Number of Trials	Outcome	RR
Xu [66]	*n*: 980,008	21	Ovarian cancer risk	1.02; CI, 0.89–1.16, *p* = 0.81
Zhou [67]	*n*: 72,275	10	Risk of breast cancer	1.04; CI 0.85–1.29, *p* = 0.68) And: 0.99; CI 0.91–1.07, *p* = 0.73) for coadministration of vitamin D and calcium
Goulão [68]	*n*: 18,808	30	Cancer Incidence	1.03; 95% CI: 0.91, 1.15) and cancer-related deaths RR: 0.88; 95% CI: 0.70, 1.09.
Bjelakovic [69]	*n*: 50,623	18	Cancer occurrence	RR 1.00; CI 0.94 to 1.06 *p* = 0.88

RR: Relative Risk.

## Data Availability

No applicable.
